# What Outcomes Are Associated with Learning About Colonialism and Its Impacts on Indigenous Peoples in Health Professional Programs? A Critical Integrative Review

**DOI:** 10.5334/pme.1407

**Published:** 2024-12-27

**Authors:** Carolyn M. Melro, Kimberly Matheson, Amy Bombay

**Affiliations:** 1 Faculty of Health, Dalhousie University, Halifax, Nova Scotia, Canada; 2 Department of Neuroscience at Carleton University and the Culture & Gender Mental Health Research Chair jointly held at The Royal’s Institute of Mental Health Research and Carleton University, Ottawa, Ontario, Canada; 3 Department of Neuroscience at Carleton University, Carleton University, Ottawa, Ontario, Canada

## Abstract

**Background:**

The Truth and Reconciliation Commission of Canada called upon health professional programs to teach about historical and on-going colonalism. Since these calls to action, there has been an increase in educational opportunities on the topic. Although it is generally assumed that learning about colonialism will reduce racism and improve allyship towards Indigenous Peoples, an evaluation of this assumption is needed.

**Purpose:**

An integrated review of the literature was conducted to assess how participation in educational experiences is associated with learner outcomes and how they may vary according to course design considerations including the guiding framework, content foci, mode of delivery, activities, and duration.

**Methods:**

Studies assessing outcomes of educational activities related to the legacy of colonialism identified in a previous scoping review, as well as any such studies published since then were included in the present study. Data synthesis was performed using content analysis of the results and discussions presented in the included papers.

**Results:**

A review of 15 papers identified a backfire effect that was only evident among the studies that included a delayed post-evaluation timeframe. In two educational experiences, it was found that learners were more likely to express unfavourable attitudes towards Indigenous Peoples post-training. These educational opportunities were designed using a cultural safety framework and followed a similar course delivery (e.g., viewing of vodcasts, use of case studies) and provided similar content (historical policies, Indigenous cultural beliefs and practices).

**Conclusions:**

The findings should be interpreted with caution but point to plausible implications related to the backfire effect of educational opportunities on learners’ attitudes towards Indigenous Peoples post-training.

In Canada and elsewhere, content on Indigenous Peoples has been absent in educational curriculum in primary, secondary, and post-secondary levels, including within health professional education programs. Research with future and current healthcare professionals suggests that most report being ill-equipped and lack confidence to provide effective culturally safe care for Indigenous Peoples [1,2] and many expressed common stereotypes and blaming attitudes [3,4]. The consequences for health inequities were highlighted in inquiries such as those into the deaths of Brian Sinclair and Joyce Echaquan who were ignored and “waited to death” because of racism and discrimination while in need of urgent care at Canadian hospitals [5,6]. In 2015, the Truth and Reconciliation Commission (TRC) called for health professional schools to teach about Indigenous health issues, including the history and legacy of residential schools and colonialism [7]. Since the call to action, our recent scoping review revealed that content related to Indigenous Peoples and the impacts of colonialism is increasingly being included within health professional training programs in Canada and elsewhere [8].

Although promoting anti-racism was a clear goal in calling for such curriculum, evaluations of student outcomes focused largely on changes in basic knowledge about Indigenous Peoples’ inequities and experiences accessing health care. Only a minority of studies evaluated attitudes towards Indigenous Peoples, such as emotional reactions, interest in working with Indigenous Peoples, and views on the educational experience [8]. Building off this earlier work to move beyond a summary of what outcomes were evaluated, the current review reports findings regarding learner outcomes of such educational initiatives from the articles identified in our scoping review and in any additional relevant articles that have been published since [9]. It was further assessed whether these outcomes vary according to the guiding theoretical framework used to develop the educational opportunity, the specific content or activities included, and/or the duration of the program.

The literature related to the inclusion of content about Indigenous Peoples and the legacy of colonialism in health professional programs reveals varying theoretical frameworks as guiding curriculum development. Education related to Indigenous Peoples in health professional programs has often focused on raising cultural awareness of different health perspectives, such as comparing and drawing differences between an Indigenous holistic wellness model and the Euro-Western biomedical model. While attention to Indigenous cultural knowledge and practices can certainly be valuable for current and future health care providers [10,11], when presented alone, it risks perpetuating cultural or explanatory stereotypes as to why Indigenous Peoples experience inequities [12] rather than focusing on how Euro-Western systems continue to oppress Indigenous Peoples. Considering that such causal explanations of health inequities have been linked with increased racist attitudes in certain contexts [13], this may have the opposite of the intended effect of reducing racist attitudes in learners. Although there are conceptual frameworks emphasizing teaching about the social and structural determinants of health [14,15], dispelling false beliefs [16,17], and creating cultural awareness [18], there are no empirical frameworks (known to the authors) to guide the development of content and learning activities to challenge commonly held false beliefs and negative social attitudes in relation to Indigenous or other populations.

Along with the different guiding frameworks, there is heterogeneity within health professional education literature in relation to the specific content, delivery methods, and duration of curriculum or training related to Indigenous Peoples. This makes it hard to make conclusive statements about how to effectively teach about Indigenous Peoples in ways that result in a reduction of racist beliefs, attitudes, and behaviours among health professionals, and improved health care that is culturally safe. This is particularly important as research in various contexts related to training that aims to reduce intergroup bias, and promote diversity, equity, and inclusion generally shows that, at best they do not work, and at worst, they exacerbate bias or negative social attitudes [19]. Content within health professional programs that teach about the legacy of historical and on-going aspects of colonialism in relation to well-being among Indigenous Peoples often comprises standalone and brief modules [8] that provide learners with limited exposure to content that might challenge and correct deep-seated beliefs and negative attitudes. It has been suggested that curricula focused on Indigenous Peoples should include opportunities for critical reflection [4], highlight Indigenous Peoples’ voices [20], and provide interactive and experiential learning opportunities [21]. Educational opportunities that provide accurate information may be helpful in dispelling false beliefs and reducing prejudicial attitudes, at least in the short term [15,16]. However, long-term evaluations are required to determine lasting outcomes, as short-term changes can revert to original beliefs and attitudes over time due to learners’ constant exposure to negative messages among peers, family and media [15].

This paper provides an integrated review of the literature to assess how participation in educational experiences that teach about the legacy of colonialism are associated with learner outcomes such as knowledge and intergroup attitudes, and how they may vary according to design considerations including the guiding framework, content foci, mode of delivery, activities, and duration. Despite the repeated calls for content on Indigenous Peoples within health professional programs, we currently know little about the variables that might influence short- and long-term outcomes such as changes in knowledge about and attitudes towards Indigenous Peoples. Given our previous work and the gap in the literature on what works, why and how when it comes to designing educational opportunities [8,9], an integrative review was chosen as it is focused on thoughtful, in-depth, and critically reflective engagement with ideas [22,23]. Building off our foundational work, this research aims to assess outcomes of learning about colonialism and whether they vary according to the guiding theoretical framework used to develop the educational opportunity, the specific content or activities included, and/or the duration of the program [22,23].

## Methodology/Methods

Studies assessing outcomes of educational activities related to the legacy of colonialism identified in a previous scoping review [8], as well as any such studies published since then [9] were included in the present study to identify learner outcomes and how they might be associated with commonly used theoretical frameworks. The original scoping review identified 14 of the 15 articles included in this integrative review, which was undertaken using the Joanna Briggs Institute (JBI) Guideline for scoping reviews [24]. The data set included English peer-reviewed journal articles published since 2000 that reported on any short and/or long-term outcome of educational activities in Canada, the United States, Australia, and New Zealand that included content related to Indigenous Peoples’ experiences with historical and on-going colonialism within post-secondary or higher education in health and social service programs. These four countries (i.e., Canada, the United States, Australia, and New Zealand) were chosen given their similar historical colonial context and common political and legal frameworks to address health inequities and social determinants of health of Indigenous Peoples. Searches of six databases and hand-searching techniques of Indigenous journals and forward and backward searching of included studies were completed in 2021 and rerun in 2023 (with a date filter of 2021 to 2023), locating 2977 and 255 articles, respectively. These articles were independently screened by two reviewers, resulting in 14 articles that met our inclusion criteria (see Melro et al. [8] for details). Any articles published since the scoping review were also included (Melro et al., [9]), which resulted in a total of 15 studies in this integrative review.

Data synthesis was performed using content analysis of the results and discussions presented in the included papers following Whittemore and Knalf’s outline that primary sources be re-ordered, coded, characterized into themes, and brought together into a cohesive and integrated summary [23]. The content analysis was inductive and conducted by the primary researcher with discussion and refinement by co-authors. For a detailed review of the theoretical frameworks, course content, evaluation approach and post-intervention outcomes commonly assessed see our scoping review [8]. The research team consists of a non-Indigenous PhD who conducted this research as part of their doctoral studies (lead author), and a non-Indigenous and Indigenous academic faculty members in a Department of Neuroscience. All authors have experience conducting research and/or teaching about the historical and ongoing effects of colonialism concerning health and social outcomes among Indigenous Peoples. We engaged in critical reflexivity throughout the process by holding regular discussions to examine commonalities across papers with similar findings. This approach allowed us to better understand how course delivery, content focus, and duration might influence reported outcomes. By reflecting on these elements, we aimed to mitigate potential biases and ensure a more comprehensive analysis.

## Findings

### Outcomes of learning about colonialism and its impacts on Indigenous peoples

#### Short term outcomes

Overwhelmingly, research on the effects of educational trainings focused on the short-term outcomes assessed immediately post educational opportunities (12 out of the 15 studies). Despite applying a range of theoretical frameworks (i.e., transformative learning theory, critical reflection and culturally informed theories) to guide the design of such educational curriculum, studies that evaluated outcomes immediately following the intervention reported positive changes associated with self-reported knowledge, beliefs and attitudes towards Indigenous Peoples [25,26,27,28]. For example, the learners that took part in a KAIROS Blanket Exercise overwhelmingly strongly agreed that it enhanced their knowledge of Indigenous Peoples’ history in Canada (99%) and that the exercise gave them a greater awareness of the impact of colonization (98%; [26]). Just under half of learners (48%) highlighted the importance of learning about this history to gain a greater understanding of Indigenous cultural contexts. Learners discussed how the activity encouraged them to critically reflect on their biases and stereotypes regarding Indigenous Peoples, with many having a new recognition of their social responsibility for working towards Indigenous health equity [26].

One community clinical immersive experience that included content on the impact of historical trauma on healthy lifestyles, well-being, and social justice elicited changes of scores on the White Racial Identity Attitude Scale, suggesting that learners’ awareness of their role in creating and maintaining oppressive systems, and the need for them to act responsibly by dismantling systemic racism through a framework of power and privilege increased as a result of the intervention. These quantitative findings align with qualitative analyses of learners’ reflective journals, which often conveyed recognition of privilege, suspension of judgement, reframing the situation and cultural consciousness [25]. Similarly, Hunt and colleagues (2015) found that teaching about the historical, political, and social aspects of Indigenous health taught in relation to contemporary healthcare issues of cultural competence, cultural safety, racism, equity and access resulted in a decrease in negative attitudes, and increased scores on knowledge, interest, and confidence in working with Australian Indigenous Peoples.

#### Long term outcomes

Only two educational opportunities included a delayed post-evaluation timeframe, both of which resulted in positive effects that dissipated over time. Thackrah and Thompson (2013) found that after a tutorial, learners’ attitudes towards Indigenous Peoples increased in a favourable direction on an Attitude Thermometer, but did not reach statistical significance [28]. Similar to others, they found an increase in favourable attitudes and knowledge about Aboriginal history, culture, and health and a heightened awareness of the role played by structural factors on health outcomes immediately following an intervention [28]. Yet, when followed up a year or two after the educational experience, the positive shifts in attitudes and knowledge among midwifery learners were not sustained [29,30]; to the contrary, relative to baseline, one year following the intervention, there was a decline in knowledge about Indigenous issues and a significant drop in positive attitudes towards Indigenous Peoples, and this decline was more pronounced two years after participation in the course. These findings suggest that the influence of these interventions was lost even before learners entered professional or clinical practice, at least in relation to these specific outcomes. As noted by others, this could be due to the limited integration of Indigenous content across the program [8], and/or to contradictory hidden curricula within health professional programs [31,32]. However, this was the only educational intervention in the scoping review to assess long-term outcomes, and so information was limited. When we compared the Thackrah and colleagues’ studies [29,30] to our own course evaluation study [9], we found a similar backfire effect three months after the course. Indeed, in one out of three cohorts, learners were more inclined to express blaming attitudes and their support for government social action and policy declined. Although, these findings were not significantly different among learners enrolled in the two other cohorts they demonstrated a similar trend. It was also found that learners who were less willing to recognize the role of historical factors on health inequities were more likely to blame Indigenous Peoples for their inequities. Those who had greater recognition of the historical factors indicated stronger support for government action or policies to address such inequities and a lower inclination to express blaming attitudes [9].

### Comparison between theoretical framework, content, delivery and long-term outcomes

When reviewing the educational opportunities that evaluated outcomes with a delayed post-evaluation timeframe and demonstrated a backfire effect on learners attitudes [9,29,30], we noted similarities in the theoretical frameworks applied, the content, and delivery. These interventions were designed using a cultural safety theoretical framework and followed a similar course delivery (e.g., viewing of vodcasts or videos prepared specifically for the unit featuring diverse Indigenous speakers, use of case studies). Both focused on providing content on family structures, historical policies, Indigenous cultural beliefs and practices, and specific professional practice issues. In addition, they both emphasized the creation of safe spaces while teaching, although an evaluation of perceived student safety in the course was not reported. One area in which the two educational opportunities differed that could influence learner’s outcomes was in the length of the programming. In Thackrah and colleague’s studies [28,29,30], the educational tutorial was 24 hours long (2 hours/12 tutorials) whereas in the Melro and colleagues [9] the course was only 9 hours (1.5 hrs/6 tutorials). Due to the breadth of content covered in both studies, we were unable to determine which factor (i.e., theoretical framework, course delivery, content, pedagogy) influenced the evaluative outcomes, if any.

## Discussion

In view of the complexity of evaluating educational opportunities within health professional programs, there is wide variation in the degree of rigour applied in program development and evaluation. However, the teaching of knowledge, skills, and attitudes forms the pedagogical framework for most health professional education programs. Within the literature, there was an overreliance on the evaluation of short-term outcomes that showed favorable changes in learners’ attitudes toward Indigenous Peoples, but studies that applied a long-term evaluation (i.e., 3-month or 1 or 2-years) found no change of attitudes compared to baseline [29,30], or worse, a greater inclination to express blaming attitudes [9]. Our team found similar results based on preliminary analyses of post-intervention responses to an educational opportunity created for federal public servants within an Indigenous serving ministry. The result of these analyses suggests that public servants that participated in an online 6-hour asynchronous training intervention that provided a brief history of colonialism in Canada and how certain aspects of colonialism serve as a root cause of ongoing health and social inequities were more likely to blame Indigenous Peoples for the inequities immediately post-educational opportunity [33].

Given the potential for a backfire effect that is counter to the educational goals of such interventions, there is a need for the development of robust and critical evaluations that explore changes in health professional learners’ beliefs, attitudes, and behaviours to determine effective different trainings that vary according to content, duration, approaches, theoretical frameworks, and pedagogy. Indeed, the studies reviewed tended to use indirect or vague language in describing the course objectives, design considerations, and theoretical frameworks. Publication biases favouring evaluations with statistically significant findings or favourable outcomes (i.e., positive changes in attitudes) mean that it is possible that the effectiveness of the educational opportunities has been overestimated in some cases, or those that found a backfire effect were unlikely to be in the published literature.

Due to the possibility of such backfire effects, educators and researchers must shift from relying on measuring knowledge-based outcomes to monitoring the effects of their educational opportunity on learners’ beliefs and negative attitudes. For educators, this may help identify attitudinal and ideological foci that are amendable to change. This shift might be achieved by using a realist evaluation approach that allows researchers and educators to develop a more sophisticated understanding of what works, why and how, including recognition that desired outcomes would take time, and as such, a need for longitudinal follow-up. In so doing, we can begin to identify viable solutions to addressing anti-Indigenous racism and inequities in health care.

## References

[1] Kutob RM, Bormanis J, Crago M, Harris JM Jr, Senf J, Shisslak CM. Cultural competence education for practicing physicians: lessons in cultural humility, nonjudgmental behaviors, and health beliefs elicitation. Journal of Continuing Education in the Health Professions. 2013 Jun; 33(3): 164–73. 24078364 10.1002/chp.21181

[2] Isaacson M. Clarifying concepts: Cultural humility or competency. J Prof Nurs. 2014 May; 30(3): 251–8. DOI: 10.1016/j.profnurs.2013.09.011 24939335

[3] Browne AJ, Fiske JA. First Nations women’s encounters with mainstream health care services. West J Nurs Res. 2001 Mar; 23(2): 126–47. DOI: 10.1177/019394590102300203 11272853

[4] Allan B, Smylie J. First Peoples, second class treatment: The role of racism in the health and well-being of Indigenous peoples in Canada. Wellesley Institute; 2015.

[5] Cecco L. Canada: outcry after video shows hospital staff taunting dying Indigenous woman. The Guardian. 2020.

[6] Geary A. Ignored to death: Brian Sinclair’s death caused by racism, inquest inadequate, group says. CBC News. 2017 Sep 18.

[7] Truth, Reconciliation Commission of Canada. Final Report of the Truth and Reconciliation Commission of Canada, Volume One: Summary: Honouring the Truth, Reconciling for the Future. Ottawa, ON: Truth and Reconciliation Commission of Canada; 2015.

[8] Melro CM, Landry J, Matheson K. A scoping review of frameworks utilized in the design and evaluation of courses in health professional programs to address the role of historical and ongoing colonialism in the health outcomes of Indigenous Peoples. Adv Health Sci Educ. 2023 Oct; 28(4): 1311–31. DOI: 10.1007/s10459-023-10217-y 37067638

[9] Melro CM, Matheson K, Bombay A. Beliefs around the causes of inequities and intergroup attitudes among health professional students before and after a course related to Indigenous Peoples and colonialism. BMC Med Educ. 2023 Apr 22; 23(1): 277. DOI: 10.1186/s12909-023-04248-7 37085777 PMC10121421

[10] Hadjipavlou G, Varcoe C, Tu D, Dehoney J, Price R, Browne AJ. “All my relations”: experiences and perceptions of Indigenous patients connecting with Indigenous Elders in an inner-city primary care partnership for mental health and well-being. Cmaj. 2018 May 22; 190(20): E608–15. DOI: 10.1503/cmaj.171390 29789285 PMC5962391

[11] Varcoe C, Browne AJ, Ford-Gilboe M, Dion Stout M, McKenzie H, Price R, et al. Reclaiming Our Spirits: Development and Pilot Testing of a Health Promotion Intervention for Indigenous Women Who Have Experienced Intimate Partner Violence. Research in Nursing & Health. 2017 Jun; 40(3): 237–54. DOI: 10.1002/nur.21795 28431458 PMC6586042

[12] Ly A, Crowshoe L. ‘Stereotypes are reality’: Addressing stereotyping in Canadian Aboriginal medical education. Med Educ. 2015; 49(6): 612–22. DOI: 10.1111/medu.12725 25989409

[13] Fourie MM, Moore-Berg SL. We cannot empathize with what we do not recognize: Perceptions of structural versus interpersonal racism in South Africa. Front Psychol. 2022 Sept 28; 13: 838675. Epub 20220928. DOI: 10.3389/fpsyg.2022.838675 36248600 PMC9555212

[14] Jones R, Crowshoe L, Reid P, Calam B, Curtis E, Green M, et al. Educating for Indigenous Health Equity: An International Consensus Statement. Acad Med. 2019 Apr; 94(4): 512–9. DOI: 10.1097/ACM.0000000000002476 30277958 PMC6445615

[15] Sharma M, Pinto AD, Kumagai AK. Teaching the Social Determinants of Health: A Path to Equity or a Road to Nowhere? Acad Med. 2018 Jan; 93(1): 25–30. DOI: 10.1097/ACM.0000000000001689 28445214

[16] Pedersen A, Barlow FK. Theory to social action: A university-based strategy targeting prejudice against Aboriginal Australians. Australian Psychologist. 2008 Sep; 43(3): 148–59. DOI: 10.1080/00050060802318587

[17] Pedersen A, Walker I, Paradies Y, Guerin B. How to cook rice: A review of ingredients for teaching anti-prejudice. Australian Psychologist. 2011 Mar 1; 46(1): 55–63. DOI: 10.1111/j.1742-9544.2010.00015.x

[18] Durey A. Reducing racism in Aboriginal health care in Australia: where does cultural education fit? Australian and New Zealand journal of public health. 2010 Jul; 34: S87–92. DOI: 10.1111/j.1753-6405.2010.00560.x 20618302

[19] Cox WT. Developing scientifically validated bias and diversity trainings that work: empowering agents of change to reduce bias, create inclusion, and promote equity. Management decision. 2022 Sep 5; 61(4): 1038–61. DOI: 10.1108/md-06-2021-0839 37090785 PMC10120861

[20] Mills K, Creedy DK, West R. Experiences and outcomes of health professional students undertaking education on Indigenous health: A systematic integrative literature review. Nurse education today. 2018 Oct 1; 69: 149–58.Epub 20180731. PubMed PMID: 30081248. DOI: 10.1016/j.nedt.2018.07.014 30081248

[21] Beavis AS, Hojjati A, Kassam A, Choudhury D, Fraser M, Masching R, Nixon SA. What all students in healthcare training programs should learn to increase health equity: Perspectives on postcolonialism and the health of Aboriginal Peoples in Canada. BMC medical education. 2015 Dec; 15: 1–1. PubMed PMID: 26400722; PubMed Central PMCID: PMC4581088. DOI: 10.1186/s12909-015-0442-y 26400722 PMC4581088

[22] Greenhalgh T, Thorne S, Malterud K. Time to challenge the spurious hierarchy of systematic over narrative reviews? Eur J Clin Invest. 2018 Jun; 48(6): e12931. Epub 2018 Apr 16. DOI: 10.1111/eci.12931 29578574 PMC6001568

[23] Whittemore R, Knafl K. The integrative review: updated methodology. Journal of advanced nursing. 2005 Dec; 52(5): 546–53. DOI: 10.1111/j.1365-2648.2005.03621.x 16268861

[24] Peters MD, Godfrey CM, Khalil H, McInerney P, Parker D, Soares CB. Guidance for conducting systematic scoping reviews. Int J Evid Based Healthc. 2015 Sep; 13(3): 141–6. Epub 2015/07/03. DOI: 10.1097/xeb.0000000000000050 26134548

[25] Alexander-Ruff JH, Kinion ES. Developing a cultural immersion service-learning experience for undergraduate nursing students. J Nurs Educ. 2019 Feb; 117–20. DOI: 10.3928/01484834-20190122-11 30721314

[26] Herzog LS, Wright SR, Pennington JJ, Richardson L. The KAIROS Blanket Exercise: Engaging Indigenous ways of knowing to foster critical consciousness in medical education. Medical Teacher. 2021 Dec 2; 43(12): 1437–43. DOI: 10.1080/0142159X.2021.1956679 34369238

[27] Hunt L, Ramjan L, McDonald G, Koch J, Baird D, Salamonson Y. Nursing students’ perspectives of the health and healthcare issues of Australian Indigenous people. Nurse education today. 2015 Mar 1; 35(3): 461–7. DOI: 10.1016/j.nedt.2014.11.019 25499968

[28] Thackrah RD, Thompson SC. Confronting uncomfortable truths: receptivity and resistance to Aboriginal content in midwifery education. Contemporary Nurse. 2013 Dec 1; 46(1): 113–22. DOI: 10.5172/conu.2013.46.1.113 24716769

[29] Thackrah RD, Thompson SC. Applying a midwifery Lens to indigenous health care delivery: the contribution of campus learning and rural placements to effecting systemic change. Canadian Journal of Nursing Research. 2018 Dec; 50(4): 179–88. DOI: 10.1177/0844562118771829 29726715

[30] Thackrah RD, Thompson SC, Durey A. Exploring undergraduate midwifery students’ readiness to deliver culturally secure care for pregnant and birthing Aboriginal women. BMC Med Educ. 2015; 15. DOI: 10.1186/s12909-015-0360-z PMC4428011 25885286

[31] Benbassat J. Undesirable features of the medical learning environment: a narrative review of the literature. Adv Health Sci Educ. 2013 Aug; 18(3): 527–36. DOI: 10.1007/s10459-012-9389-5 22760724

[32] Melro CM. What Works, Why, and How? The Effect of Educational Interventions on Health Professional Learners’ Causal Beliefs and Attitudes Towards Indigenous Peoples’ Experiences with Historical and Ongoing Colonialism [Doctoral dissertation]. Halifax: Dalhousie University; 2023. 237.

[33] Melro, CM, Bombay, A. Summary report: Indigenous Service Canada’s Pilot Program on Understanding the root causes of health and social inequities between Indigenous and non-Indigenous (Settler) people in Canada. [unpublished report]. 2021 Jun.

